# Premature Menarche Associated with Primary Hypothyroidism in a 5.5-Year-Old Girl

**DOI:** 10.1155/2011/678305

**Published:** 2011-08-23

**Authors:** Dhrubajyoti Sharma, Devi Dayal, Anju Gupta, Akshay Saxena

**Affiliations:** ^1^Departments of Pediatrics, Advanced Pediatrics Center, Postgraduate Institute of Medical Education and Research, Chandigarh-160012, India; ^2^Departments of Radiodiagnosis, Advanced Pediatrics Center, Postgraduate Institute of Medical Education and Research, Chandigarh-160012, India

## Abstract

Children with hypothyroidism generally have delayed pubertal development. Rare association with precocious puberty may occur especially in long standing untreated patients. The cardinal features of hypothyroidism induced pseudo precocious pubertal development include thelarche, galactorrhea and menarche. Other characteristics features are an absence of sexual hair and retardation of linear growth. Its manifestation as isolated menarche has been rarely reported. Recently, a five and half year old girl presented to us with history of one episode of vaginal bleeding. A pelvic ultrasonogram revealed multiple cysts in both ovaries and subsequent investigations led to a diagnosis of autoimmune hypothyroidism.

## 1. Case Report

A five- and half-year-old girl presented with history of one episode of vaginal bleeding seven days prior to admission. The amount of bleeding was small, noticed by mother as blood spots on undergarments. There was no history of local trauma or discharge, foreign body insertion, bleeding from any other site, and difficulty in micturition. There was no history to suggest prior central nervous system insult, raised intracranial pressure, polyuria, polydipsia, or ingestion of hormonal preparations. She was the second child born at term to a low socioeconomic status family. Her physical and mental development was said to be normal by the parents. There was no family history of a thyroid disorder or precocious puberty.

On examination, child appeared pale. There was no edema, goiter, or lymphadenopathy. Her weight and height were 17.2 kilograms and 96.5 centimeters, respectively, (both below 3rd percentiles according to CDC growth charts). Head circumference was 51 centimeters. Heart rate was 90 per minute and blood pressure 90/70 mm Hg. Breast and pubic hair development was Tanner stage 1. Abdomen was protuberant without organomegaly or palpable mass. Neurological and ophthalmologic examinations were normal. A thorough gynaecological examination revealed normal genitalia for age and ruled out local trauma or foreign body. Menstrual bleeding (menarche) was considered after excluding local causes. In view of short stature, an initial impression of isosexual precocity caused by acquired hypothyroidism was made.

**Figure 1:**
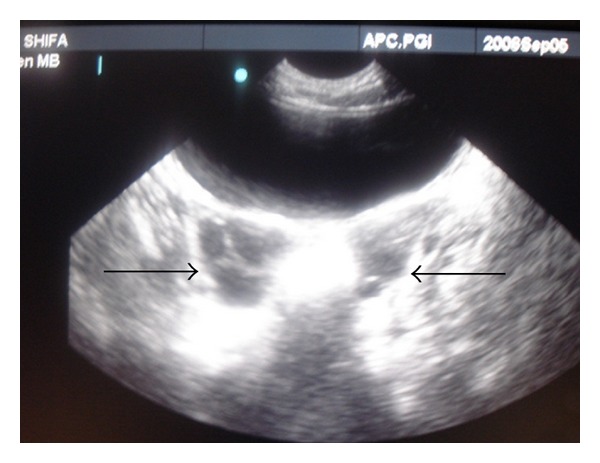
Ultrasonogram showing multicystic ovaries (Rt > Lt).

Investigations revealed hemoglobin of 8.8 g/dL, total leukocyte count 6800/mm^3^, platelet count 1,50,000/mm^3^, and macrocytic hypochromic anemia on peripheral smear examination. Thyroid function tests showed gross hypothyroidism with total triiodothyronine (*T*_3_) 0.45 pg/mL (normal, 0.7–2 pg/mL), total thyroxine (*T*_4_) 32 ng/mL (normal, 55–135 ng/mL), and thyroid-stimulating hormone (TSH) 133.4 mIU/mL (normal, 0.17–4 mIU/mL). Follicle stimulating hormone (FSH) was 10 mIU/mL (normal, 1.0–4.2 mIU/mL), Luteinizing hormone (LH) 0.5 mIU/mL (normal, 0.02–0.18 mIU/mL), estradiol (*E*_2_) 127.58 pg/mL (normal, 6–27 pg/mL), and prolactin (PRL) 26.30 ng/mL (normal, 3–24 ng/mL). Antithyroid microsomal antibodies (anti-TMA) determined by semiquantitative microtiter particle agglutination method were strongly positive (3+). Her bone age corresponded to 3 years. A pertechnetate thyroid scan revealed normal sized lobes with adequate tracer uptake. Thyroid ultrasound showed mild reduction in echogenicity and normal volume for age. Pelvic ultrasonography revealed normal sized uterus and enlarged ovaries (right ovary 5.6 cc and left 5.5 cc). Both ovaries had multiple cysts with largest measuring 1.2 cms diameter on right side and 1.2 × 1 cms on left side (Figure 1).

Levothyroxine (5 *μ*g/kg/d) therapy was initiated. There was no recurrence of vaginal bleeding till the first follow-up visit one and half months after hospital discharge. Repeat hormonal estimations were normal (*T*_3_ 2.05 ng/mL, *T*_4_ 131.9 ng/mL, TSH 2.42 mIU/mL, FSH 1.27 mIU/mL, LH 0.352 mIU/mL, *E*_2_ 16.4 pg/mL, and PRL 18 ng/mL). However, ultrasonogram done after 3 months of thyroxine therapy still showed multiple cysts in the right ovary and single cyst in left one (right ovary 3 cc and left ovary 2 cc). Repeat sonography a month later demonstrated absence of cysts. She has remained symptoms-free and showed a catch-up height increase of 9 centimeters over 1 year followup.

## 2. Discussion

Delayed pubertal development is a common manifestation of long standing untreated hypothyroidism. Precocious puberty may occur rarely as the presenting feature [1]. The onset is with thelarche with or without galactorrhea followed by menarche with absence of hair development as a characteristic feature. Another clinical clue to hypothyroidism in these cases is the decreased linear growth, quite opposite of what is seen in patients with true precocious puberty [1]. Isolated menarche, as seen in our patient, is an extremely rare presentation of hypothyroidism-related precocity [2]. Only a few cases have been reported so far. The typical clinical clues of short stature and delayed bone age were present in our case, so diagnosis of hypothyroidism could be easily entertained after excluding local causes of bleeding. In addition, important manifestations that were reported previously and detected in our case as well were bilateral ovarian enlargement with multiple cysts, hyperprolactinemia, and increased levels of gonadotropins, mainly FSH and E_2_ [2–4]. This association of precocious pubertal development and hypothyroidism, although seemingly unphysiologic, may occur in almost 50% of children with severe hypothyroidism of long duration [1]. The cause of hypothyroidism in these patients is often undiagnosed lymphocytic thyroiditis although it may be seen in association with congenital hypothyroidism also [5]. In our patient, the bone age delay and skeletal retardation indicated that the pathology began at about 3 years of age. Since autoimmune thyroiditis is uncommon before 6 years of age, we performed a thyroid scan that excluded dysgenesis or ectopia, the usual congenital causes of thyroid decompensation during growing years. Presence of anti-TMA favoured a diagnosis of autoimmune thyroiditis.

The underlying hormonal mechanisms in hypothyroidism-associated precocity remain obscure. Wyk and Grumbach tried to explain precocious puberty by an overlap in negative feedback regulation with overproduction of gonadotropins as well as thyrotropin (both share common *α* subunit) in response to thyroid deficiency [6]. But although gonadotropins are elevated in these patients, these have been observed to be GnRH unresponsive or bioinactive in earlier studies [7]. Also advancement of skeletal maturation characteristically associated with elevated gonadotropin states is not seen. So, gonadotropin excess does not appear to cause sexual precocity associated with hypothyroidism. The FSH and *E*_2_ levels were elevated in our patient although the clinical presentation with short stature, delayed BA and loss of consonance of pubertal development along with laboratory evidence of severe primary hypothyroidism indicated hypothyroidism-induced pseudoprecocity. We however could not do GnRH test to definitely exclude gonadotropin-dependent precocious puberty. 

Another theory is that extreme TSH elevation seen in profound hypothyroidism induces FSH like-effects on the gonads resulting in multicystic ovaries, uterine bleeding, and breast enlargement. Hence, precocious puberty secondary to hypothyroidism behaves like an incomplete form of gonadotropin-dependant precocious puberty [8]. According to prolactin theory, hyperprolactinemia as a result of chronic stimulation of TRH enhances the sensitivity of ovaries to even trace amounts of circulating gonadotropins prepubertally [8]. Although PRL increase in the index patient was only mild, the increased sensitivity of ovaries to gonadotropins along with extremely elevated TSH having FSH-like effects could have caused multicystic ovaries.
